# Hemocompatible LAT1-inhibitor can induce apoptosis in cancer cells without affecting brain amino acid homeostasis

**DOI:** 10.1007/s10495-020-01603-7

**Published:** 2020-05-13

**Authors:** Magdalena Markowicz-Piasecka, Johanna Huttunen, Ahmed Montaser, Kristiina M. Huttunen

**Affiliations:** 1 Laboratory of Bioanalysis, Department of Pharmaceutical Chemistry, Drug Analysis and Radiopharmacy, Medical University of Lodz, ul. Muszyńskiego 1, Lodz, 90-151 Poland; 2 School of Pharmacy, Faculty of Health Sciences, University of Eastern Finland, P.O. Box 1627, Kuopio, 70211 Finland

**Keywords:** l-type amino acid transporter 1 (LAT1), Inhibitor, Apoptosis, Hemocompatibility, Amino acid homeostasis

## Abstract

Increased amounts of amino acids are essential for cancer cells to support their sustained growth and survival. Therefore, inhibitors of amino acid transporters, such as l-type amino acid transporter 1 (LAT1) have been developed. In this study, a previously reported LAT1-inhibitor (KMH-233) was studied for its hemocompatibility and toxicity towards human umbilical vein endothelial cells (HUVEC) and human aortic smooth muscle cells (AoSMCs). Furthermore, the cytotoxic effects against human breast adenocarcinoma cells (MCF-7) and its ability to affect mammalian (or mechanistic) target of rapamycin (mTOR) and nuclear factor kappa-light-chain-enhancer of activated B cells (NF-κB) signaling were evaluated. Moreover, the effects of this inhibitor to modulate LAT1 function on the cell surface and the brain amino acid homeostasis were evaluated after intraperitoneal (i.p.) administration of LAT1-inhibitor (23 µmol/kg) in mice. The results showed that LAT1-inhibitor (KMH-233) is hemocompatible at concentrations below 25 µM and it does not affect coagulation in plasma. However, it can reduce the total protein amount of mTOR and NF-κB, resulting in increased apoptosis in LAT1-expressing cancer cells. Most importantly, the inhibitor did not affect mouse brain levels of l-Leu, l-Tyr or l-Trp or modulate the function of LAT1 on the MCF-7 cell surface. Therefore, this inhibitor can be considered as a safe but effective anti-cancer agent. However, due to the compensative mechanism of cancer cells for their increased amino acid demand, this compound is most effective inducing apoptosis when used in combinations with other chemotherapeutics, such as protease inhibitor, bestatin, as demonstrated in this study.

## Introduction

Cancer cells require enormous amounts of nutrients to support their uncontrolled and rapid division [1, 2]. Amino acids are essential nutrients for protein synthesis and metabolism and therefore cancer cells must have increased uptake of amino acid across the plasma membrane [3]. l-Type amino acid transporter 1 (LAT1, *SLC7A5*) is a pH and sodium independent transporter carrying large, neutral, aromatic or branched essential l-amino acids (EAAs), such as l-Leu, l-Phe, l-Tyr, l-Trp, l-His, l-Met, l-Ile, and l-Val into the cells [4, 5]. It functions as an antiporter and exchanges an intracellular EAAs or glutamine, for the external ones with 1:1 stoichiometry [6]. LAT1 functions also together with alanine-serine-cysteine transporter 2 (ASCT2; *SLC1A5*) that facilitates the uptake of glutamine back into the cells [7]. The light chain of LAT1 is linked via a disulfide bond to a CD98 heavy chain (4F2hc; *SLC3A2*), which assists in the transport of amino acids but also aids in trafficking the protein complex to the cell membrane [8].

LAT1 is highly expressed in the brain and placenta but to a lesser extent also in other tissues [5]. However, LAT1 is upregulated in a variety of cancer and their metastases, including the brain, breast, colorectal, gastric, head and neck, kidney, liver, lung, ovarian, pancreatic, prostate and thyroid cancers, to name few [9–11]. Moreover, high LAT1 expression has been associated with a significantly shorter survival of patients and poorer prognosis in the case of breast, and prostate cancers [12, 13].

**Fig. 1:**
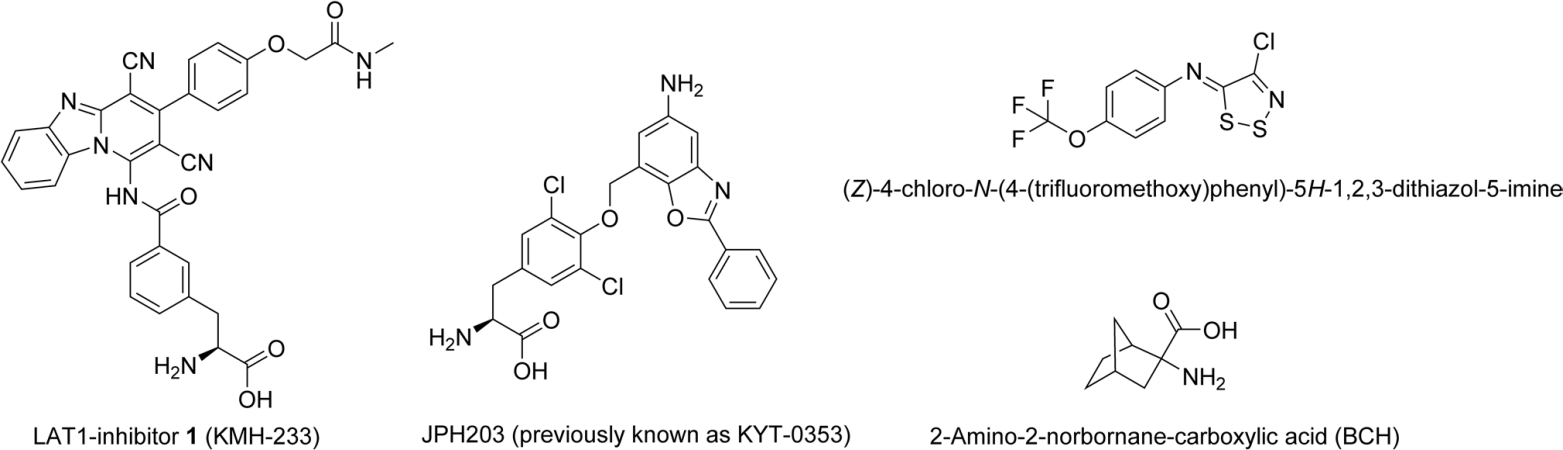
Chemical structures of LAT1-inhibitor **1** (KMH-233), JPH203, 1,2,3-dithiazole and BCH

Therefore, LAT1 has gained a growing interest not only as a therapeutic target for anticancer therapy but also as a diagnostic target for tumor imaging or as a cancer biomarker [14]. Inhibition of l-Leu uptake, an amino acid that is known to promote protein synthesis via mammalian (or mechanistic) target of rapamycin (mTOR) pathway, has been reported to result in antiproliferative effects [10]. This has been achieved by a LAT1-inhibitor, JPH203 (Fig. 1) resulting in reduced proliferation of several cancer cell lines [15–20]. The compound has also been studied in Phase I clinical trials [11]. In addition to JPH203, other LAT1-binding compounds have also been studied as potential cancer chemotherapeutics (Fig. 1). These include non-transportable blockers of LAT1 that are structural thyronine derivatives of JPH203 [21], irreversible LAT1-inhibitors 1,2,3-dithiazoles [22], and 2-amino-2-norbornane-carboxylic acid (BCH), which has been referred as a system l inhibitor in the literature [23–25], although it acts more like a competing LAT1-substrate rather than LAT1-inhibitor [11, 26]. Moreover, from these compounds, BCH is a non-selective substrate of the system l family, having a binding affinity for all its members, including LAT1, LAT2 (*SLC7A8*), LAT3 (*SLC43A1*), and LAT4 (*SLC43A2*) [10].

We have earlier reported a LAT1-selective slowly reversible inhibitor (KMH-233, compound **1** in Fig. 1) that can potentiate antiproliferative efficacy in breast cancer cells together with cisplatin and bestatin [27]. However, the hemocompatibility of this inhibitor and the effects on coagulation, as well as the detailed cytotoxic effects of this LAT1-inhibitor have not yet been thoroughly studied in the past. Besides, although the effects of LAT1 inhibition on the EAA levels in the tumor microenvironment have been considered [11, 17], the effects on the normal function of the organs expressing LAT1 have not been studied thoroughly so far. This is a really important aspect, since e.g., in the brain LAT1 is responsible for delivering EAA to neurons and glial cells and therefore, focusing on only the efficacy of LAT1 inhibition in the tumor microenvironment may neglect the possible central nervous system (CNS) side effects of the chemotherapy. Therefore, the ability of compound **1** to affect the brain amino acid homeostasis and LAT1 function on the plasma membrane is studied and reported herein.

## Materials and methods

### Cell cultures and other materials

Cell culturing ingredients for human umbilical vein endothelial cells (HUVEC; RRID CVCL_2959; Lonza, Italy; Cat. No. CC-2517) were as follows: medium EGM-2 - medium + bullet kit (Lonza, Clonetics, Italy), accutase (Sigma Aldrich, Germany), HEPES buffered saline solution (Lonza, Italy). Human primary Aortic Smooth Muscle Cells (AoSMC) (ScienCell, US) were cultured using Dulbecco’s Phosphate-Buffered Saline (DPBS, Gibco), accutase (Sigma Aldrich, Germany), and smooth muscle cell medium (ScienceCell, US). Before seeding the flasks were precoated with Poly-l-Lysine (10 mg/mL; Sciencell, US). MCF-7 human breast adenocarcinoma cells (HTB-22; RRID CVCL_0031) were purchased from the European Collection of Authenticated Cell Culture (ECACC, Salisbury, UK, Cat. No. 86012803), and were cultured in standard conditions (37 ºC, 5% CO_2_) using Dulbecco’s modified Eagle medium (DMEM, Gibco, Thermo Fisher Scientific, US) supplemented with l-glutamine (2 mM, Gibco, Thermo Fisher Scientific, US), heat-inactivated fetal bovine serum (10%, Gibco, Thermo Fisher Scientific, US), penicillin (50 U/mL, Gibco, Thermo Fisher Scientific, US), and streptomycin (50 µg/mL, Gibco, Thermo Fisher Scientific, US).

All reagents and solvents used in other analytical studies were commercial and high purity of analytical grade or ultra-gradient LCMS-grade purchased from Sigma (St. Louis, MO, USA), J.T. Baker (Deventer, The Netherlands), Merck (Darmstadt, Germany), Riedel-de Haën (Seelze, Germany) or Thermo Fisher Scientific (Waltham, MA, USA). Water was purified using a Milli-Q Gradient system (Millipore, Milford, MA, USA).

### Basic coagulation tests

Basic coagulation studies were conducted using Bio-Ksel (Poland) reagents; Bio-Ksel System APTTs reagent and calcium chloride, Bio-Ksel PT plus reagent (thromboplastin and solvent), and thrombin (3.0 UNIH/mL). Triton X-100 used in the erythrotoxicity test was obtained from Polish Chemical Reagents (Poland). Coagulation parameters, including prothrombin time (PT), international normalized ratio (INR), partially activated thromboplastin time (APTT) and thrombin time (TT) were determined in the presence of the compound **1** according to the routine diagnostic procedure using coagulometer (CoagChrom-3003 Bio-Ksel, Poland) described previously [28, 29]. The results were presented as mean ± SD, n = 5.

### Red blood cell lysis assay

The protocol of red blood cell (RBC) lysis assay was published previously [28]. The test compound was incubated with 2% RBC suspension for one hour at 37 ºC. Control samples with 0.9% NaCl (spontaneous control) and Triton-X (positive control) were also conducted. The amount of released hemoglobin was measured spectrophotometrically at 550 nm (Cecil 2021, UK). Positive control constituted 100% of hemolysis. The results are expressed as a percentage of released hemoglobin, n = 4. The effect of compound **1** on RBC morphology was evaluated using a phase-contrast Opta-Tech inverted microscope (Opta-Tech, Poland), at 400-times magnification, equipped with software (OptaView 7, Opta-Tech, Warsaw, Poland) for image analysis.

### HUVEC and AoSMC cells viability

HUVEC and AoSMC were subcultured according to the manufacturers’ (Lonza, Italy, and Sciencell, US, respectively) guidelines. The viability of HUVEC and AoSMC cells was assessed using the WST-1 assay (Takara, Takara Bio Europe, France), which is based on the reaction of cleavage of tetrazolium salts by mitochondrial dehydrogenase in viable cells, according to the protocol published previously [30]. The cells were seeded at the density of 7500 (HUVECs) and 5000 (AoSMCs) per well on 96-well plates. The cells were cultured for 24 h to obtain 70% confluency, following by co-treatment with the studied compound [1] at the concentration of 0.1–200 µM for 24 h (37 ºC, 5% CO_2_). After 24-hour incubation, the compound solutions were discarded of all wells, and the cells were washed with culture medium at the volume of 100 µL, followed by the addition of the WST-1 reagent dissolved in cell culture medium (100 µL per well). The plates were incubated at 37 ºC with 5% CO_2_ for 2 h and the absorbance was read at 450 nm using microplate reader (iMARK, Bio-Rad, Bio-Rad Laboratories Inc., US). The results are expressed as a percentage of the control samples treated with pure medium, which constituted 100% viability. The data are presented as mean ± standard deviation (SD), n = 6–8. The morphology of HUVECs and AoSMCs were examined microscopically using an inverted microscope with phase contrast (magnification 100x) (software OptaView 7, Opta-Tech, Warsaw, Poland).

### Flow cytometric apoptosis analysis

Apoptosis was examined using FITC Annexin V Apoptosis Detection Kit with propidine iodide (PI) (Cat. No. 640914, Biolegend, UK) and cell staining buffer (Cat. No. 420201, Biolegend, UK). MCF-7 cells were seeded at the density of 60 000 per well on 24 well plates, following by 24-hour incubation (37 ºC, 5% CO_2_). Then the medium was replaced by 250 µL of fresh medium (control wells) or medium with tested compounds at appropriate concentrations corresponding to IC_50_ values [27], and the cells were incubated for the next 72 h. Afterward, the cells were harvested, collected to Eppendorf tubes and centrifuged (1200 rpm, 5 min). The cell pellets were suspended in cold cell staining buffer (BioLegend, UK) and washed twice with this solution. Then the cells were resuspended in 100 µL of binding buffer (BioLegend, UK), and solutions of propidine iodide (PI) (10 µL) and FITC-Annexin (5 µL) were added. The samples were vigorously mixed, and incubated for 20 min at room temperature in the dark, followed by the analysis conducted on the cytometer (CytoFlex, blue laser, 480 nm, BeckMan Coulter, US). The results were analyzed using Kaluza 2.1 BeckMan Coulter software. The analysis was made on the principle that annexin V(−) and PI (−) cells were considered as living cells (E–), annexin V (+) and PI (−) as early-apoptotic cells (E-+), annexin V(+) and PI (+) as late-apoptotic cells (E++), and annexin V (−) and PI (+) as necrotic cells (E−+). The results are presented as the percentage of the cells collected in gate B.

### Total amounts of mTOR and NF-κB

Enzyme-Linked Immunosorbent Assay (ELISA) kits were used (mTOR SimpleStep ELISA Kit and *NFκB* p65 Total SimpeStep ELISA Kit, Abcam, Cambridge, UK) to quantify mammalian (or mechanistic) target of rapamycin (mTOR) and nuclear factor kappa-light-chain-enhancer of activated B cells (NF-κB) amounts, respectively. The studied compounds (100 µM of compound **1**, 5 mM l-Leu or 10 µM rapamycin) were incubated for 0.5–96 h with MCF-7 at a density of 2 × 10^5^ in 6-well plates. The control wells were treated with the solvent only (0.5% DMSO). Cells were solubilized using the provided extraction buffer with the kit, incubated on ice for 20 min and centrifuged at 18,000 rpm for 20 min at 4 ºC. The supernatants were stored at − 80 ºC until the day of the analysis. Standards and samples were then analyzed following the manufacturer protocol (ELISA sandwich method) and by reading the absorbance with the Envision plate reader (EnVision, Perkin Elmer, Waltham, MA, USA) at 450 nm. The results were analyzed and presented as pmol of formed mTOR or NF-κB per ml.

### Brain amino acid homeostasis

Adult male mice weighing 25 ± 5 g were supplied by Envigo (Venray, Netherlands). Mice were housed in stainless steel cages on a 12 h light (07:00–19:00) and 12 h dark (19:00–07:00) cycle at an ambient temperature of 22 ± 1 ºC with a relative humidity of 50–60%. All experiments were carried out during the light phase. Tap water and food pellets (Lactamin R36; Lactamin AB, Södertälje, Sweden) were available ad libitum. Compound **1** (1.36 mM) was dissolved in a vehicle containing 10% (v/v) of DMSO, 20% (w/v) of hydroxypropyl-β-cyclodextrin and 0.9% (w/v) NaCl in water. A dose of 23 µmol/kg of compound **1** was given as a bolus injection (i.p.) to mice. The mice were decapitated at selected time points between (10–480 min) and brain tissues for sample preparation to be analyzed by liquid chromatography-mass spectrometric (LC-MS/MS) analysis.

Tissue samples were weighed and homogenized with ultrapure water (1:3). 100 µL of the homogenates was taken, and the proteins were precipitated with 300 µL of acetonitrile containing the internal standard (labetalol). Samples were vortexed and centrifuged at 14,000 rpm at 4 ºC for 10 min. 200 µL of supernatant was mixed with 100 µL of ultrapure water and injected to LC-MS/MS (Agilent 1200 Series Rapid Resolution LC System (Agilent Technologies, Waldbronn, Germany), together with Agilent 6410 Triple Quadrupole Mass Spectrometer equipped with an electrospray ionization source ((Agilent Technologies, Palo Alto, CA., USA).

The amounts of three known LAT1-utilizing amino acids, l-Leu, l-Tyr, and l-Trp were quantified with a method described earlier [31]. Briefly, an Acquity UPLC BEH Amide column (100 mm × 2.1 mm, 1.7 µm; Waters Corporation, Milford, MA, USA) was used at a flow rate 0.3 mL/min with gradient elution of eluents consisting of 20 mM ammonium formate in H_2_O:ACN (1:1; A) and 20 mM ammonium formate in H_2_O:ACN (1:9; B), pH 3. Mass spectrometric detection was performed with multiple reaction monitoring at the positive mode with the following transitions: 182→164.9 for l-Tyr, 132.1→86.1 for l-Leu, 205.1→188 for l-Trp, and 329→294 for labetalol. Data were acquired using the Agilent MassHunter Workstation Acquisition software (Data Acquisition for Triple Quadrupole Mass Spectrometer, version B.03.01) and processed and analyzed with Quantitative Analysis (B.04.00) software. The lower limit of quantification (LLOQ) for the amino acids in tissue samples was 0.1 nM. The methods were linear, selective, accurate and precise over the calibration range of 0.25–2500 nM.

### LAT1 cellular function

MCF-7 cells were carefully washed with pre-warmed HBSS (Hank’s balanced salt solution) containing 125 mM choline chloride, 4.8 mM KCl, 1.2 mM MgSO_4_, 1.2 mM KH_2_PO_4_, 1.3 mM CaCl_2_, 5.6 mM glucose, and 25 mM HEPES (pH 7.4 adjusted with 1 M NaOH) after removal of the culture medium. Pre-incubation was done with 500 µL of pre-warmed HBSS at 37 ºC for 10 min before adding the substrate for the uptake experiments. The ability of compound **1** to inhibit a known LAT1 substrate, [^14^C]-l-leucine, the cells were incubated with 100 µM of compound **1** in HBSS or medium (250 µL) for 10 min, 60 min or 24 h at 37 ºC. The incubation buffer was removed and the cells were incubated with 0.76–75 µM (0.25 mCi/ml) of [^14^C]-l-leucine (PerkinElmer, Waltham, MA, USA) for 5 min, in uptake buffer (HBSS, 250 µL). After incubation, the transport was stopped by adding 500 µL of ice-cold HBSS and the cells were washed two times with ice-cold HBSS. The cells were then lysed with 250 µL of 0.1 M NaOH (60 min) and the lysate was mixed with 1.0 mL of Emulsifier safe cocktail (Ultima Gold, PerkinElmer, Waltham, MA, USA). The radioactivity in the cells was measured by liquid scintillation counting (MicroBeta^2^ counter, PerkinElmer Waltham, MA, USA) and the amount of [^14^C]-l-leucine in cell lysates was calculated from the standard curve that was prepared by spiking known amounts of [^14^C]-l-leucine to cell lysates and normalized to protein concentration. The protein concentrations on each plate were determined as a mean of three samples by Bio-Rad Protein Assay, based on the Bradford dye-binding method, using bovine serum albumin (BSA) as a standard protein and measuring the absorbance (595 nm) by multiplate reader (EnVision, Perkin Elmer, Inc., Waltham, MA, USA).

### Ethical issues

The studies on the biological material were approved by the Bioethics Committee of the Medical University of Lodz (RNN/109/16/KE). The procedure for the preparation of red blood cells for erythrotoxicity and plasma for coagulation studies were described previously [28, 29]. The experimental procedures involving animals were made in compliance with the European Commission Directives 2010/63/EU and 86/609, and approved by the Institutional Animal Care and Use Committee of the University of Eastern Finland (Animal Usage Plan number ESAVI/3347/04.10.07/2015). All efforts were made to minimize the number of animals used and to minimize their suffering.

### Data analysis

All statistical analyses were performed using GraphPad Prism v. 5.03 software (GraphPad Software, San Diego, CA., USA). Statistical differences between groups were tested using one-way ANOVA, followed by a Tukey’s multiple comparison test and presented as mean ± SD, with significant difference denoted by *P < 0.05, **P < 0.01, ***P < 0.001.

## Results

### Basic coagulation parameters

**Fig. 2:**
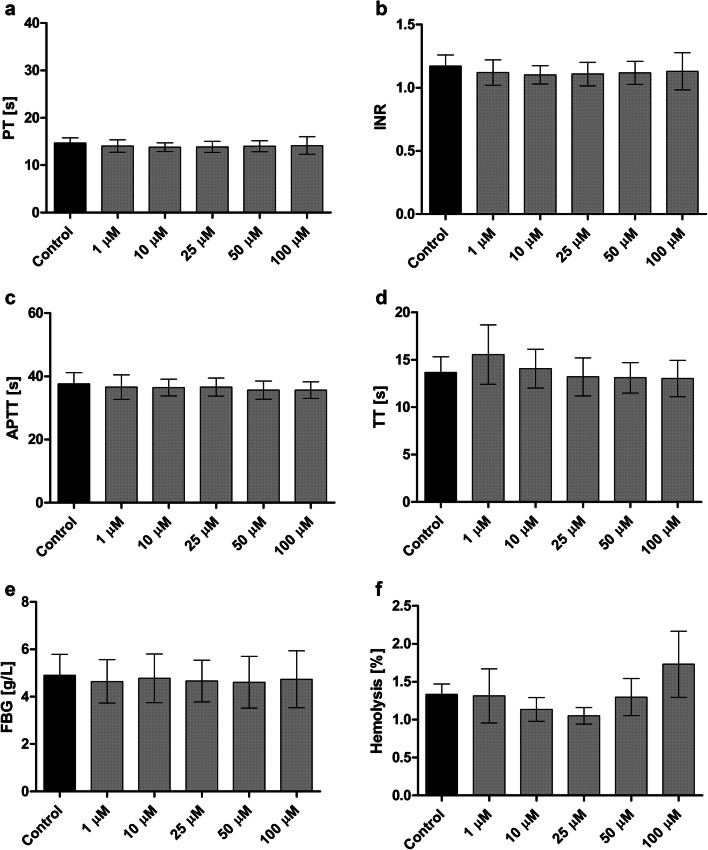
The effects of 1–100 µM LAT1-inhibitor **1** on basic coagulation parameters in human plasma;** a** Prothrombin Time (PT),** b **International Normalized Ratio (INR), **c** Partially Activated Thromboplastin Time (APTT), **d** Thrombin Time (TT), **e** fibrinogen concentration (FBG) or **f** the integrity of erythrocyte membrane expressed as the hemolysis rate. The data are expressed as mean ± standard deviation (SD), n = 4–5

The effects of 1–100 µM LAT1-inhibitor **1** on basic coagulation parameters were studied with an array of different studies in human plasma. These results showed that the exposure to compound **1** did not significantly affect the value of prothrombin time (PT), or international normalized ratio (INR) (Fig. 2a, b). The results of partially activated thromboplastin time (APTT) measurements revealed that the compound did not influence the intrinsic coagulation pathway either (Fig. 2c). Besides, the compound did not alter the process of fibrin polymerization (constant thrombin time; TT) (Fig. 2d) nor the concentration of fibrinogen (FBG) (Fig. 2e).

### Red blood cell lysis assay and morphology

**Fig. 3:**
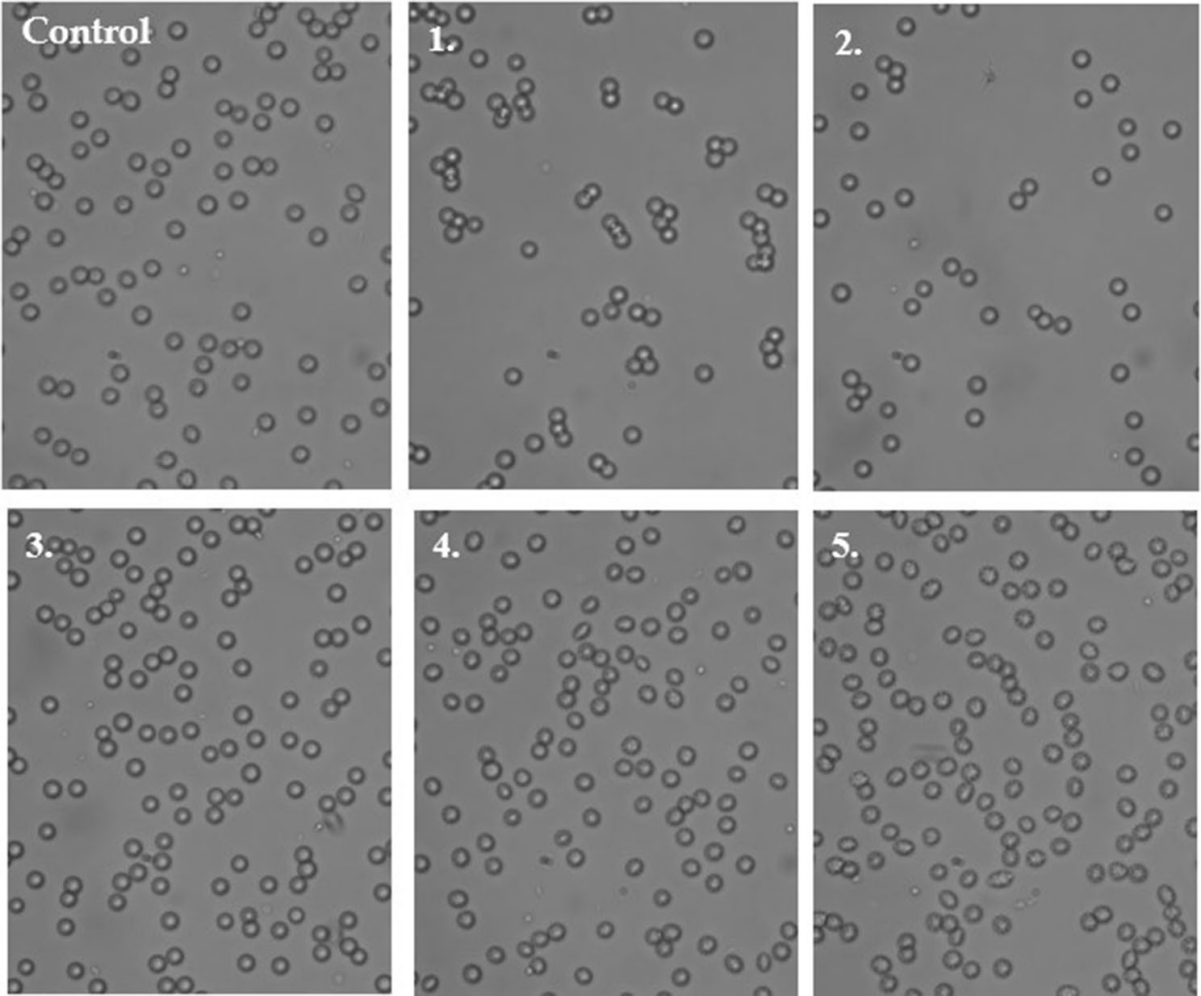
The effects of compound **1** (1, 10, 25, 50 and 100 µM, respectively) on erythrocyte morphology. Representative phase-contrast images are shown (magnification of 400 times)

The effects of LAT1-inhibitor **1** on RBC hemolysis were also studied with a lysis assay. The compound did not exert unfavorable effects on erythrocytes over the studied concentration range (1–100 µM) (Fig. 2f). The microscopic analysis of erythrocyte morphology (Fig. 3) showed also that compound **1** did not affect the morphology of erythrocytes at the concentration below 25 µM. However, at 50 µM concentration single echinocytes were observed and at 100 µM concentration the compound induced extensive echinocytosis.

### Human umbilical vein endothelial cell and human aortic smooth muscle cell viability

**Fig. 4:**
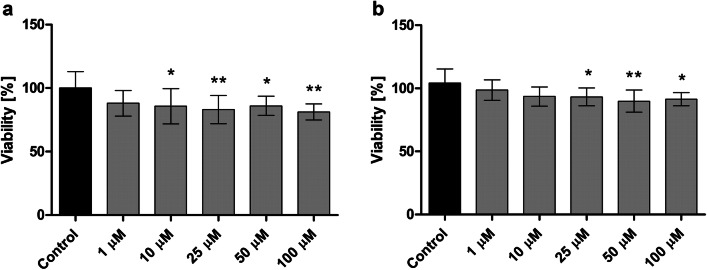
The effects of 1–100 µM LAT1-inhibitor **1** on the viability of ** a** HUVEC and ** b ** AoSMC cells after 24 h incubation, the results are presented as mean ± SD (n = 8) and an asterisk denotes a statistically significant difference from the respective control (*P < 0.05, **P < 0.01, one-way ANOVA, followed by Tukey’s test)

**Fig. 5:**
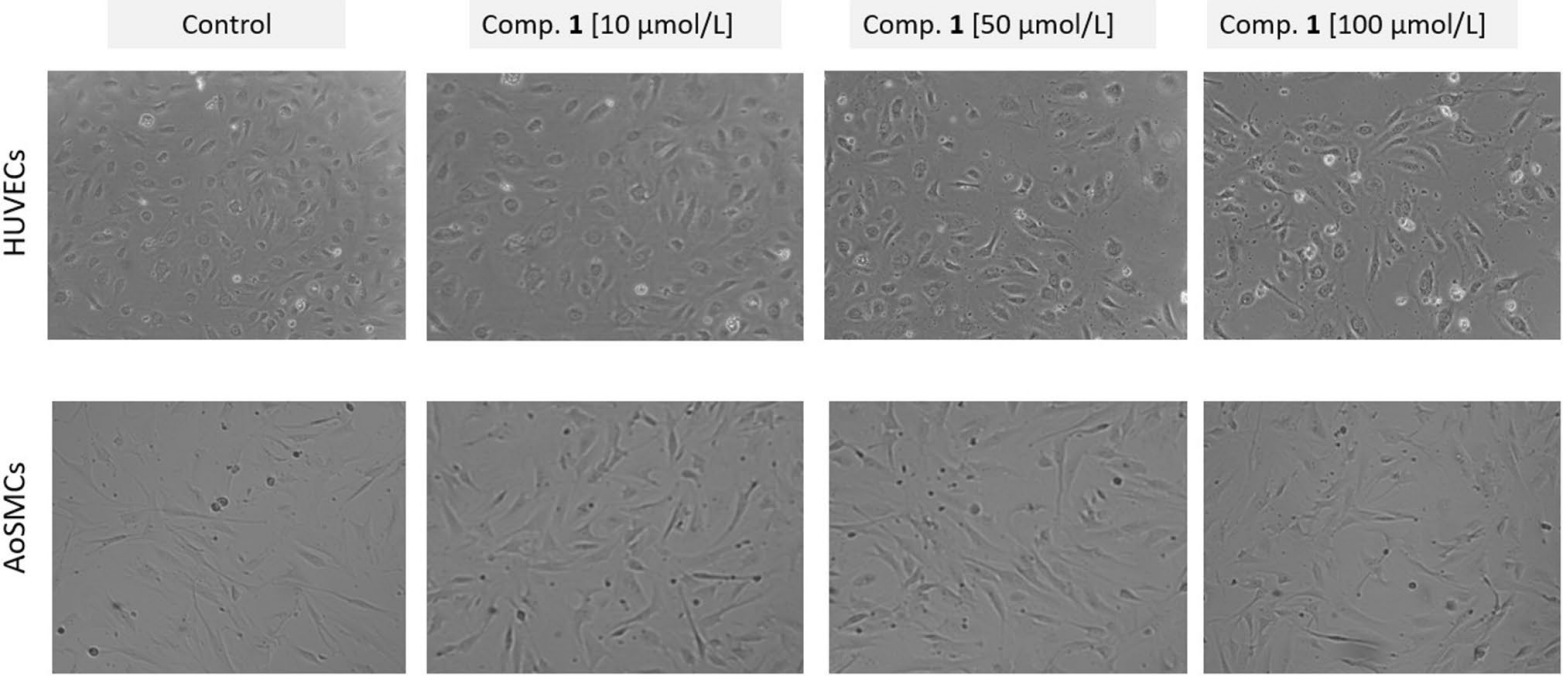
The effects of 1–100 µM LAT1-inhibitor **1** on endothelial cell (HUVECs) and smooth muscle cell (AoSMC) viability, representative phase-contrast cell images are shown after 24 h of incubation (100-fold magnification)

The effects of LAT1-inhibitor **1** on cell viability of HUVEC and AoSMC were studied by using WST-1 assay. The analysis of both HUVEC and AoSMC viability showed similar effects caused by the compound **1**, and the viability of cells depended on the concentration of the compound (Fig. 4). The highest concentration of compound **1** (100 µM) contributed to the small but significant decrease in cellular viability; 19% decreased with HUVECs and 9% decreased with AoSMCs. At the higher concentrations (50–100 µM) the compound also contributed to the increased number of rounded, dead cells (Fig. 5). However, at lower concentrations (1–25 µM), the studied compound did not affect the morphology of HUVECs or AoSMCs.

### Flow cytometric apoptosis analysis

**Fig. 6:**
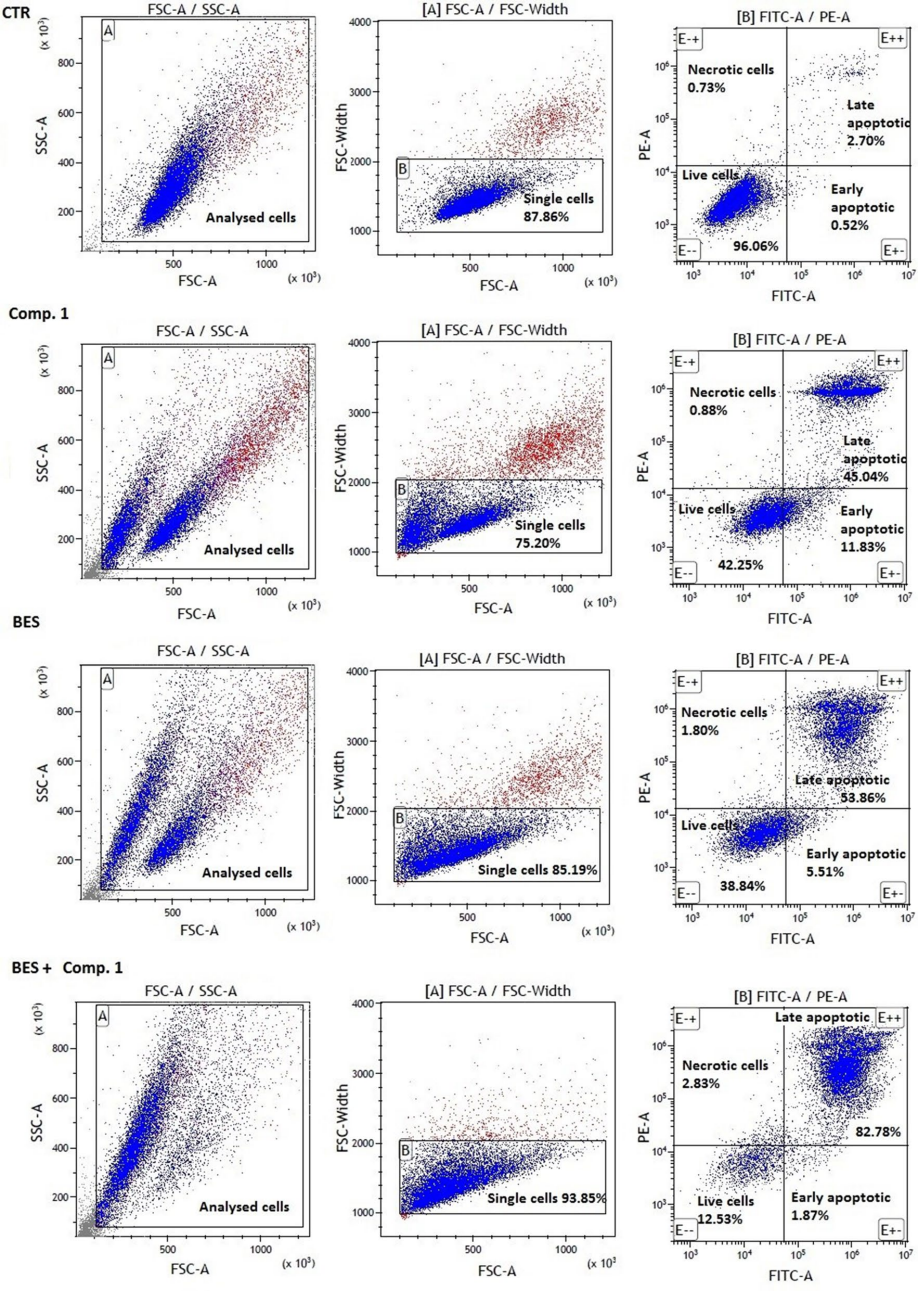
The effects of 100 µM LAT1-inhibitor **1** and 250 µM bestatin (BES) alone (second and third line, respectively) and in combination (fourth line) on viability and apoptosis of MCF-7 cells compared to the untreated control (first line)

To analyze the cytotoxic and apoptotic effects of LAT1-inhibitor **1**, the compound was incubated with MCF-7 cells for 72 h and then stained with propidine iodide (PI) and Annexin V (AV) for flow cytometry analysis (Table 1, Fig. 6). The amount of compound **1** for flow cytometric study was selected by its ability to inhibit the cell growth during 72 h incubation (IC_50_ value 100 µM) [27]. Furthermore, the effects of LAT1-inhibitor were studied in the presence of other cytotoxic compounds, aminopeptidase inhibitor, bestatin (250 µM) and platinum-based antineoplastic DNA-binder, cisplatin (25 and 50 µM), based on our previous results [27]. All the examined compounds, apart from the mixture of LAT1-inhibitor **1** and bestatin, decreased the percentage of cells gathered in gate B. Combination of LAT1-inhibitor **1** and bestatin contributed to the significant increase in the number of single cells collected in gate B. This may be explained by the greater effects of the mixture on cell aggregation, causing higher amounts of single cells. All the tested compounds and their mixtures contributed to a significant decrease in the percentage of viable cells (AV–PI -). Importantly, the addition of bestatin to LAT1-inhibitor **1** resulted in much lower cell viability (13.54 ± 1.05%) in comparison to LAT1-inhibitor **1** alone (43.03 ± 0.72%) (Table 1). Staining the cells with AV and PI showed also that all tested compounds significantly contributed to the increase in early- and late- apoptotic cells. The strongest effect regarding the induction of apoptosis exhibited the afore-mentioned mixture of bestatin and LAT1-inhibitor **1** (82.53 ± 0.79%). Curiously, the co-treatment of cisplatin and LAT1-inhibitor **1** did not contribute to the synergistic effect regarding apoptosis induction, not even at higher concentration (50 µM), although the mixture was earlier noted to be anti-proliferative [27].

**Table 1:** Annexin V-FITC/PI double staining analysis of apoptosis in MCF-7 induced by compound **1** (100 µM), bestatin (BES, 250 µM), cisplatin (CIS, 25 and 50 µM) and their combinations. The results are presented as mean ± SD (n = 3)

Compound [µmol/L]	Single cells in gate B [%]^a^	Living cells [E − −] [%]^b^	Necrotic cells [E − +] [%]^b^	Early apoptotic [E + −] [%]^b^	Late apoptotic [E + +] [%]^b^
Control	87.93 ± 0.19	95.07 ± 1.47	0.94 ± 0.28	0.85 ± 0.38	3.15 ± 0.81
Comp. 1 [100 µM]	75.67 ± 0.72*	43.03 ± 0.72*	1.08 ± 0.18	11.55 ± 0.29*	44.33 ± 0.62*
BES [250 µM]	84.31 ± 0.86*	41.35 ± 2.50*	2.15 ± 0.91	4.49 ± 1.26*	52.02 ± 1.99*
1 + BES [100 + 250 µM]	93.67 ± 0.46*, #	13.54 ± 1.05*, #	1.76 ± 0.97	2.18 ± 0.29*, #	82.53 ± 0.79*, #
CIS [50 µM]	76.43 ± 0.05*	37.72 ± 2.90*	1.52 ± 0.17	8.30 ± 0.28*	55.45 ± 2.73*
1 + CIS [100 + 50 µM]	73.03 ± 0.34*	33.83 ± 2.22*	0.63 ± 0.09	14.91 ± 1.82*	50.64 ± 3.32*
CIS [25 µmol/L]	76.36 ± 0.89*	39.83 ± 3.42*	1.10 ± 0.30	9.44 ± 0.90*	49.63 ± 2.25*
1 + CIS [100 + 25 µM]	74.23 ± 0.95*	36.71 ± 1.99*	0.57 ± 0.15	13.69 ± 1.85*	49.02 ± 0.61*

^a^Single cells gathered within the gate B reflecting the % of the absolute number of acquired events; ^b^the single cells gathered in gate B were divided depending on staining with Annexin V and PI; (E − −) living cells; (E − +) necrotic cells; (E + −) early-apoptotic cells; (E + +) late-apoptotic cells. The results are presented as mean ± standard deviation (SD), n = 3. The values given in bold represent statistically significant (p < 0.05) difference respective to the control. An asterisk (*) depicts the significant difference (p < 0.05) between the comp. ***1*** (100 µM) and ***1*** + BES (100 + 250 µM) combination and a hashtag (#) denotes the significant difference (p < 0.05) between BES (250 µM) and ***1*** + BES (100 + 250 µM) combination

### Amounts of mTOR and NF-ƘB in human breast adenocarcinoma cells

**Fig. 7:**
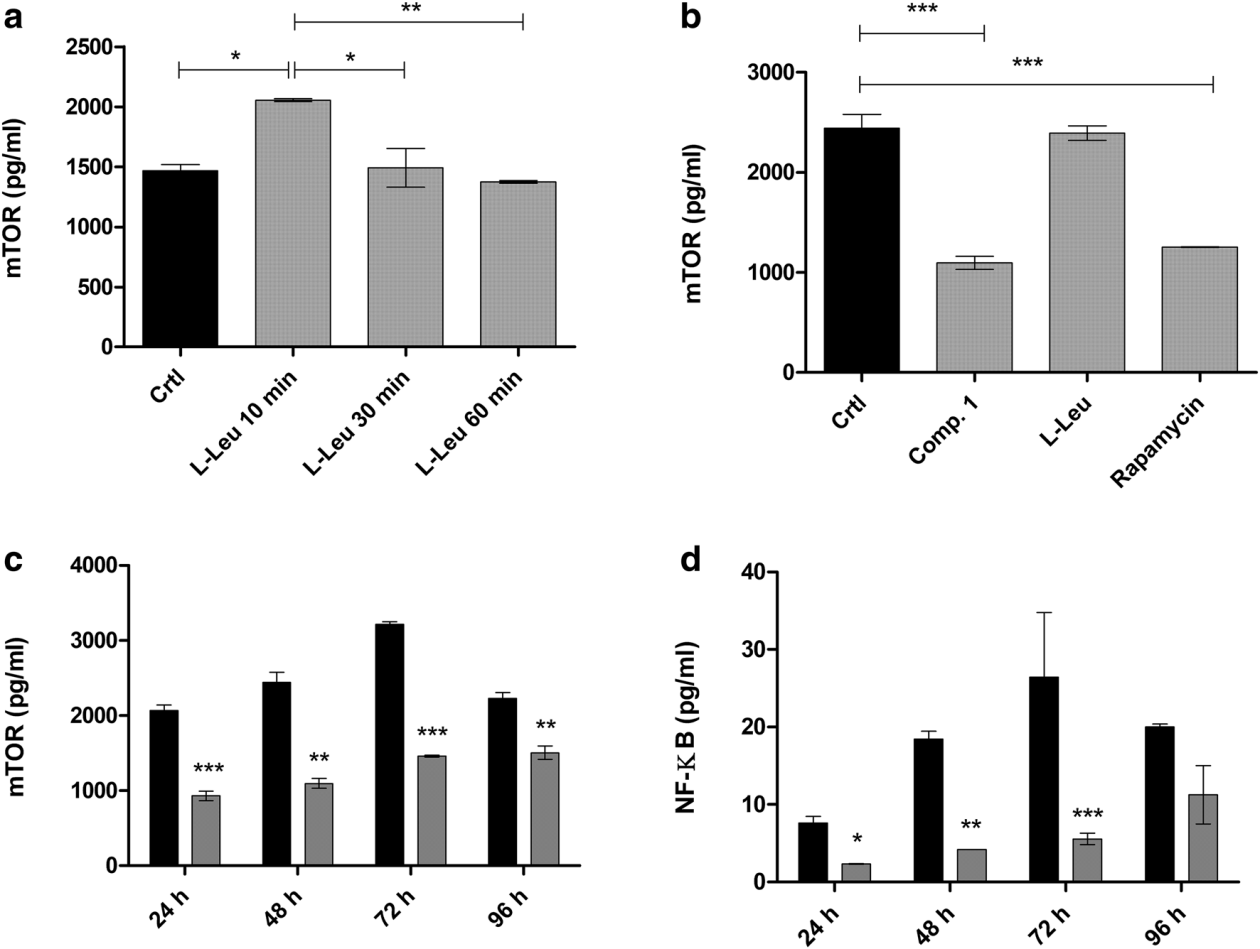
Effects of **a** 5 mM l-Leu on mammalian target of rapamycin (mTOR) total amount after 10, 30 and 60 min, **b** 100 µM LAT1-inhibitor **1**, 5 mM l-Leu and 1 µM rapamycin on mTOR total amount after 48 h incubation, **c** 100 µM LAT1-inhibitor **1** (grey bars) on mTOR total amount after incubation of 24, 48, 72 and 96 h (control represented as black bars), and **d** 100 µM LAT1-inhibitor **1** (grey bars) on the total amount of NF-ƘB after incubation for 24, 48, 72 and 96 h (control represented as black bars), data are presented as mean ± SD (n = 3) and an asterisk denotes a significant difference from the respective control (*P < 0.05, **P < 0.01, ***P < 0.001, one-way ANOVA, followed by Tukey’s test)

Since it is well known that inhibition of LAT1 suppresses mTOR signaling that is associated with the decreased growth of the cancer cells [10], the aim in this study was to evaluate if LAT1-inhibitor **1** can affect the total amount of mTOR in MCF-7 cells. Firstly, the effects of l-Leu on mTOR total protein amount in cells were studied at the time points of 10, 30 and 60 min. As seen in Fig. 7a and 5 mM l-Leu upregulated the mTOR expression with a time-dependent manner, which is in accordance with the previous reports [32, 33]. Contrarily, compound **1** at 100 µM concentration, which equals its IC_50_ value for anti-proliferative effects [27], reduced the total amount of mTOR after 48 h incubation with the cells (55.10 ± 2.64%). This was comparable to the inhibition by 10 µM rapamycin (48.68 ± 0.19%) (Fig. 7b**)**. The effect also lasted a relatively long time, since after 96 h, the total amount of mTOR was still approximately 32% smaller compared to the control one (Fig. 7c).

Today, we also know that NF-ƘB signaling pathways have a remarkable role in cancer initiation, development, metastasis, and resistance to chemotherapy [34, 35]. Moreover, mTOR is known to be a key regulator of NF-ƘB via the Akt serine-threonine kinase pathway [36]. Therefore, it was studied if LAT1-inhibitor **1** (100 µM) can also affect the total NF-ƘB protein content after 24, 48, 72 and 96 h incubation with MCF-7 cells. As seen in the Fig. 7d, the compound **1** also decreased significantly the total NF-ƘB protein content after 24–72 h (43–79%). After 96 h, the inhibitory effect started to dilute, similarly as with mTOR total protein amount.

### Brain amino acid homeostasis

**Fig. 8:**
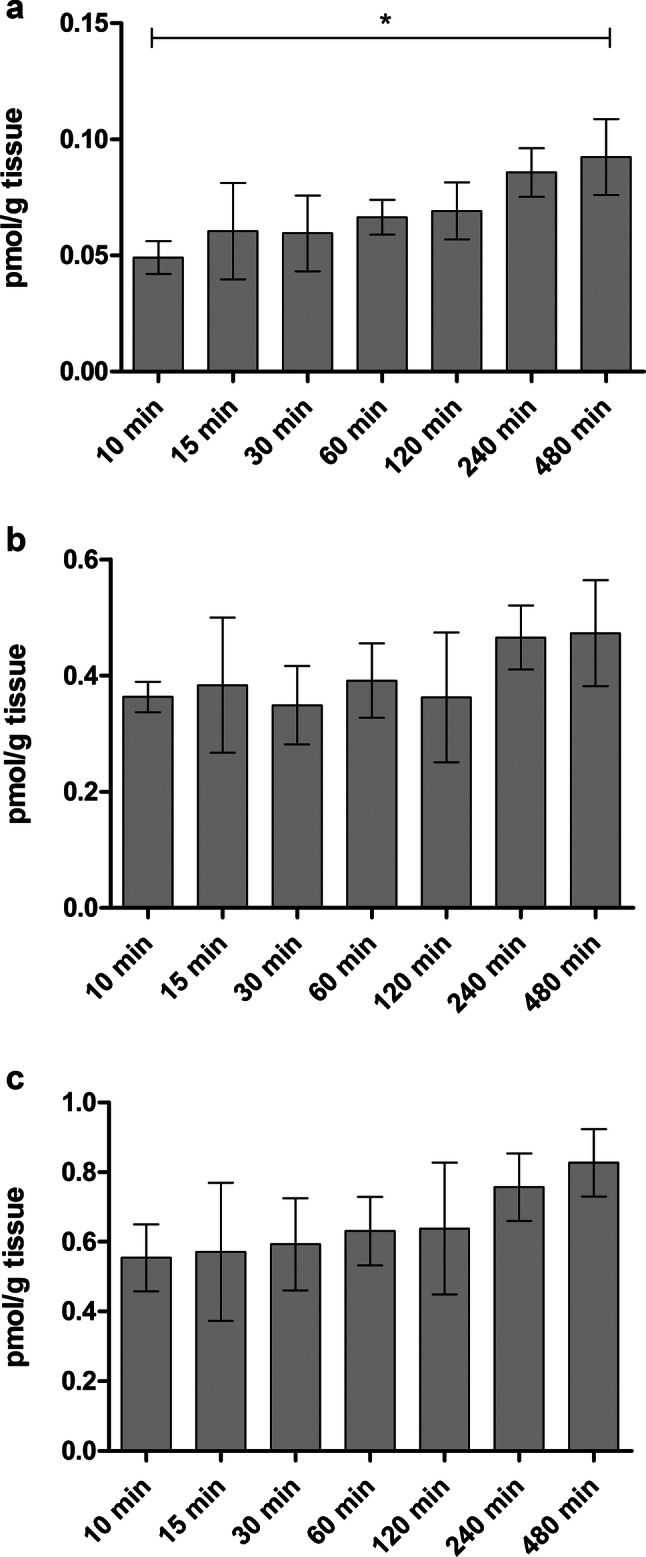
Brain amounts of l-Trp (**a**), l-Leu (**b**) and l-Tyr (**c**) over 480 min period after a single dose of compound **1** (23 µmol/kg, i.p.) to mice. The data are presented as mean ± SD (n = 3) and an asterisk denotes a significant difference from the respective control (*P < 0.05, one-way ANOVA, followed by Tukey’s test)

Since LAT1 is highly expressed on the luminal and abluminal sides of the blood-brain barrier (BBB) [37], ensuring the supply of EAAs to the neurons and glial cells, it is really important to evaluate if the developed LAT1inhibitors affect the brain amino acid homeostasis. In our previous publication, LAT1-inhibitor, KMH-233, was proved to behave reversibly; it did not cross the BBB nearly at all and it was released from the endothelial cells back to the bloodstream [27]. However, the detachment from the cell surface was noted to be relatively slow and therefore, in the present study, the effects of compound **1** to brain amino acid homeostasis were evaluated. The levels of three known LAT1-substrates, l-Leu, a promotor of mTOR-mediated energy metabolism, as well as l-Trp and l-Tyr, precursors of neurotransmitters serotonin and dopamine, respectively, were followed in the brain after a single i.p. dose of 23 µmol/kg of compound **1** to mice. As seen in Fig. 8, the followed EAA levels remained constant over the 8 h inspection period. The bigger amino acids, l-Trp and l-Tyr, showed some gradual increase over time, however, the difference between the time points was only statistically significant with l-Trp at the first (10 min) and the last (480 min) time points.

### LAT1 function on the cell surface

**Fig. 9:**
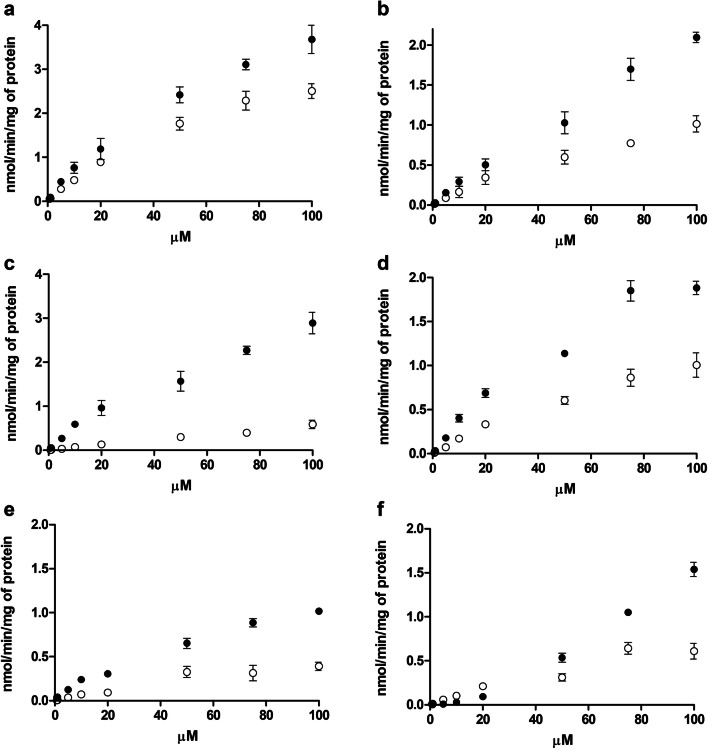
Ability of 100 µM LAT1-inhibitor **1** to inhibit 0.76-75 µM (0.25 mCi/ml) of [^14^C]-l-leucine uptake after 10 min (**a**), 60 min (**c**) and 24 h (**e**) incubation of compound **1** in HBSS and after 10 min (**b**), 60 min (**d**) and 24 h (**f**) incubation of compounds **1** in medium, data are presented as mean ± SD (n = 3)

The ability of LAT1-inhibitor **1** to modulate the LAT1 function on the cell surface was also studied with MCF-7 cells. Firstly, 100 µM compound **1** was incubated in HBSS buffer for 10 min, 60 min and 24 h incubation and then the uptake of 0.76-75 µM of [^14^C]-l-leucine (in HBSS) was measured. Since HBSS affected the cells during the longer incubation (the leucine uptake in control cells was much lower compared to 10 min incubation in Fig. 9a, c, and e), the cells were also treated with 100 µM compound **1** in DMEM medium for 10 min, 60 min, and 24 h, the medium was removed and the uptake of 0.76–75 µM of [^14^C]-l-leucine in HBSS without LAT1-inhibitor was then analyzed over 5 min period. According to these results, it was obvious that LAT1-inhibitor **1** did not induce LAT1 function on the cell surface (Fig. 9b, d, and f) since the uptake of [^14^C]-l-leucine remained on the same levels over the incubation period (10 min−24 h), V_max_ ranging from 1.72 (24 h) to 2.37 (60 min).

## Discussion

According to the basic coagulation studies and erythrocyte lysis assays, the LAT1 inhibitor **1** can be considered hemocompatible in systemic circulation at concentrations below 100 µM (Figs. 2 and 3). The compound did not affect the cell viability of HUVECs at a concentration below 10 µM or AoSMCs at a concentration below 50 µM (Figs. 4 and 5). It has been reported that growing HUVEC express to functional LAT1 [38], although it was concluded in the same publication that these results need to be interpreted very carefully since it is not the normal stage of the blood vessels. Therefore, we concluded that the LAT1-inhibitor studied herein can be considered relatively safe. A well-known LAT1-inhibitor JPH203 has already advanced to clinical trials to treat some advanced solid tumors [39]. However, there is no detailed report about how well this compound is tolerated in humans and since JPH203 is known to be transported as a substrate also by organic anion transporting polypeptides (OATP1B1, OATP1B3, and OATP2B1) and organic anion transporter 3 (OAT3) [40], it would be vital to evaluate its hepato- and nephrotoxicity. It has also reported that JPH203 is metabolized readily to its *N*-acyl derivative in rat, monkey and human liver S9 subcellular fraction. The effects of this metabolite both on inhibition of LAT1 as well as cytotoxic effects should be carefully studied in the future. Unlike JPH203, LAT1-inhibitor **1** is LAT1-selective and not have an affinity for OATPs [27]. Moreover, comparing its structure to JPH203, it does not have an aromatic amino group to be metabolized by conjugation reaction (Fig. 1). Therefore, it was found to be stable in both mouse liver microsomes in the presence of NADPH (phase I metabolism) as well as in mouse liver S9-subcellular fraction (phase II metabolism) [27].

The studied LAT1-inhibitor **1** was also able to reduce the total protein amount of mTOR and subsequently also the total protein amount of NF-ƘB in cells during 96 h incubation, indicating that it can inhibit the cell growth and survival (Fig. 7). The studies with other published LAT1-inhibitors have shown similar effects on the mTOR signaling pathway [10, 17, 21]. However, this is the first report that inhibiting LAT1 and l-Leu uptake can also result in significant inhibition of NF-ƘB signaling, either directly or via mTOR inhibition. Since NF-ƘB can stimulate cell proliferation, prevent apoptosis, regulate tumor angiogenesis, promote tumor metastasis, affect tumor metabolism and induce chemotherapy resistance [34, 35], its inhibition is highly desired property for a LAT1-inhibitor. However, the inhibitory mechanism needs to be carefully studied in the future, whether it is direct or via mTOR inhibition and with a wider range of concentrations. In this study, relatively high concentration (100 µM) was used, which was selected based on the IC_50_ value of antiproliferative effects measured after 72 h incubation [27].

The more detailed apoptosis studies with flow cytometer revealed that compound **1** at 100 µM significantly decreased the percentage of viable cells (43.03 ± 0.72%) comparing to control (95.07 ± .47%), and induced apoptosis (44.33 ± 0.62% vs. 3.15 ± 0.81%; Table 1). Importantly, the combination of compound **1** with bestatin (250 µM) contributed to the improved effects concerning apoptosis induction since the mixture led to the formation of 82.53 ± 0.79% late-apoptotic cells without changing the number of dead cells (1.76 ± 0.97% vs. 0.85 ± 0.38% for control, *p* > 0.05). Curiously, the combination of LAT1-inhibitor **1** and cisplatin did not result in synergistic apoptotic effects, although we have previously reported that compound **1** can improve the anti-proliferative effects of cisplatin against MCF-7 cells [27]. Therefore, the mechanisms of apoptosis induced by LAT1-inhibitor **1** needs to be clarified in the future. A previously synthesized LAT1 inhibitor, JPH203 at 3 mM concentration induced anti-proliferative effects and apoptosis of YD-38 cells. However, the total number of early and late apoptotic cells did not exceed 17% [15]. On the other hand, JPH203 (3 mM) induced approximately 50% apoptosis in Saos2 osteosarcoma cells, which was mediated via the mitochondrial intrinsic apoptotic pathway [18]. However, considering the affinity of JPH203 for LAT1 (IC_50_ of [^14^C]-l-leucine uptake was 1.3 µM), surprisingly high amounts were needed to exert apoptotic effects. Therefore, it raises a question, whether this compound has cytotoxic effects other than what is mediated via LAT1 inhibition and if the inhibitor is effective enough at clinically used concentrations.

Finally, the effects of LAT1 inhibitors on the normal function of the organs that express LAT1, such as the brain, have not been studied thoroughly in the past, while the effects of the LAT1-inhibition on the EAA levels in cancer cells have been widely considered [11, 17]. In this study, LAT1-inhibitor **1** was found to not affect brain amino acid homeostasis in vivo (Fig. 8). We have previously reported that the inhibitor **1** does not cross the BBB, instead, it is slowly and reversibly bound to LAT1 on the BBB [27]. Moreover, its t_max_ in mouse plasma after 23 µmol/kg, i.p. administration was 10 min and the one in the brain was 30 min. Furthermore, the compound was mainly eliminated from plasma and detached from the BBB after 120 min. Therefore, due to the irreversible manner of this inhibition, the amounts of l-Leu, l-Tyr and l-Trp, an energy metabolism signaling molecule and essential precursors for neurotransmitter production, respectively, were not reduced below the control level during 8 h study period. Furthermore, this study also shows that there are sufficient amounts of EAAs stored in the brain to maintain the EAA homeostasis despite LAT1-inhibitory treatment. However, LAT1-inhibitor **1** increased the amount of l-Trp in the time point of 480 min compared to the time points of 10 min. The same trend was also seen with l-Tyr, although it was not statistically significant due to the relatively high variation between the samples. Thus, it is possible that LAT1 expression can be upregulated by inhibiting its function. Curiously, it has been reported that JPH203 can upregulate both LAT1 and CD98 expression in vitro in chronic 120 days’ incubation of cells [41]. However, in that study, the expressions of the proteins were determined with semi-quantitative immunoblot. Therefore, in this study, the ability of LAT1-inhibitor **1** to induce the function of LAT1 on the cell surface of MCF-7 cells was also studied (Fig. 9). However, in the present study, which measured the LAT1 function as cellular uptake of [^14^C]-l-leucine, there was no evidence that shorter (10–60 min) or longer (24 h) incubation times with 100 µM compound **1** would have induced LAT1 function.

Thus, the effects of LAT1-inhibitors on LAT1 expression on the BBB as well as on cancer cells should be studied more thoroughly with more accurate quantitative proteomic methods, such as with LC-MS/MS in future to estimate the real long-term efficacy of LAT1-inhibitor treatment and the possibility of adaptive drug resistance towards the compounds. Moreover, by using relatively high amounts of LAT1-inhibitor, such as 20–30 µM that were used in the above mention in vitro study [41], one can fully inhibit LAT1 and block the EAA supply, even if its expression was upregulated. However, it is unlikely, that such huge amounts are achieved in systemic circulation and around the cancer cells without any adverse effects. Therefore, if LAT1 is upregulated with smaller clinically relevant amounts and in a long term use, there is a risk that there will not be enough LAT1-inhibitor to starve the cancer cells at the end, which may then result in insufficient antiproliferative efficacy of LAT1-inhibitor. In the previous pharmacokinetic study with LAT1-inhibitor **1**, administered as 23 µmol/kg (i.p.) to mice, the C_max_ in plasma was only approximately 19.5 nM [27], while with JPH203 dosed with 45 nmol/kg (i.v.) in rats, the C_max_ in plasma was approximately 59.07 nM [42]. Therefore, if LAT1 expression and function can be upregulated in vitro with nanomolar concentrations, there is a possibility that the LAT1 inhibitor is not sufficient to starve cancer cells with those concentrations. Therefore, cancer cells can become resistant to LAT1-inhibitor. Moreover, it is likely that when cancer cells are starved from their EAAs, the compensating mechanisms will be activated, such as pinocytosis of macromolecules and subsequent proteolytic degradation, to retain the amino acid supply. Thus, in our opinion LAT1-inhibitors are most likely not effective enough alone, if not used in combinations with other chemotherapeutics that simultaneously affect other critical targets of the cancer cells or with agents that can block the compensating amino acid supply, such as aminopeptidase inhibitor, bestatin.

Most importantly, the effects of LAT1-inhibitors on immune response in cancer should be studied very carefully in the future since it has been reported that LAT1 is expressed on activated T-cells having a major role in the transport of EAA in those cells [38, 43]. Thus, more efforts should be paid on the effects of LAT1 inhibition in a broader sense, taking into account the effects on immune cells as well as healthy organs requiring constant EAA supply, such as the brain. Moreover, the possible upregulating mechanisms of LAT1 as well as their consequences in cancer chemotherapy should be carefully evaluated before the clinical trials.

## Conclusions

In conclusion, in this study previously reported LAT1-inhibitor **1** (KMH-233) was found to be hemocompatible and relatively safe, as it induced only minor inhibitory effects on the growth of primary HUVEC and AoSMC and only at relatively high concentrations (> 10 µM). Moreover, LAT1-inhibitor **1** did not affect the brain amino acid homeostasis or modulate the function of LAT1 on the cell surface. However, it was effective to reduce mTOR and NF-ƘB total protein amounts in MCF-7 cancer cells and induced apoptosis effectively together with bestatin. Therefore, this inhibitor is considered to be most efficacious when used together with other chemotherapeutics.

## Data Availability

All data and materials used or produced in this manuscript support the findings and comply with field standards.

## Abbreviations

AoSMC: Aortic smooth muscle cells
HUVEC: Human umbilical vein endothelial cells
LAT1: l-type amino acid transporter 1
MCF-7: Human breast adenocarcinoma cells
mTOR: Mammalian (or mechanistic) target of rapamycin
NF-κB: Nuclear factor kappa-light-chain-enhancer of activated B cells
